# Integrating single-cell transcriptomics to reveal the ferroptosis regulators in the tumor microenvironment that contribute to bladder urothelial carcinoma progression and immunotherapy

**DOI:** 10.3389/fimmu.2024.1427124

**Published:** 2024-08-22

**Authors:** Ziang Chen, Jia Hu, Yuxi Ou, Fangdie Ye, Weijian Li, Shenghua Liu, Haowen Jiang

**Affiliations:** ^1^ Department of Urology, Huashan Hospital, Fudan University, Shanghai, China; ^2^ Fudan Institute of Urology, Huashan Hospital, Fudan University, Shanghai, China; ^3^ Department of Anesthesiology, The Second Affiliated Hospital of Anhui Medical University, Anhui Medical University, Hefei, Anhui, China; ^4^ National Clinical Research Center for Aging and Medicine, Huashan Hospital, Fudan University, Shanghai, China

**Keywords:** single-cell, tumor microenvironment, bladder cancer, ferroptosis, immunotherapy, prognosi

## Abstract

**Background:**

Ferroptosis, as a novel form of programmed cell death, plays a crucial role in the occurrence and development of bladder cancer (BCa). However, the regulatory mechanisms of ferroptosis in the tumor microenvironment (TME) of BCa remain to be elucidated.

**Methods:**

Based on single-cell RNA (scRNA) transcriptomic data of BCa, we employed non-negative matrix factorization (NMF) dimensionality reduction clustering to identify novel ferroptosis-related cell subtypes within the BCa TME, aiming to explore the biological characteristics of these TME cell subtypes. Subsequently, we conducted survival analysis and univariate Cox regression analysis to explore the prognostic significance of these cell subtypes. We investigated the relationship between specific subtypes and immune infiltration, as well as their implications for immunotherapy. Finally, we discovered a valuable and novel biomarker for BCa, supported by a series of *in vitro* experiments.

**Results:**

We subdivided cancer-associated fibroblasts (CAFs), macrophages, and T cells into 3-5 small subpopulations through NMF and further explored the biological features. We found that ferroptosis played an important role in the BCa TME. Through bulk RNA-seq analysis, we further verified that ferroptosis affected the progression, prognosis, and immunotherapy response of BCa by regulating the TME. Especially ACSL4+CAFs, we found that high-level infiltration of this CAF subtype predicted worse prognosis, more complex immune infiltration, and less response for immunotherapy. Additionally, we found that this type of CAF was associated with cancer cells through the PTN-SDC1 axis, suggesting that SDC1 may be crucial in regulating CAFs in cancer cells. A series of *in vitro* experiments confirmed these inferences: SDC1 promoted the progression of BCa. Interestingly, we also discovered FTH1+ macrophages, which were closely related to SPP1+ macrophages and may also be involved in the regulation of BCa TME.

**Conclusion:**

This study revealed the significant impact of ferroptosis on bladder cancer TME and identified novel ferroptosis-related TME cell subpopulations, ACSL4+CAFs, and important BCa biomarker SDC1.

## Introduction

Bladder cancer ranks among the most prevalent malignant tumors affecting the urinary system. According to statistics, since 2023, there have been nearly 500,000 new cases of bladder cancer and 200,000 deaths globally each year (1, 2). Approximately 75% of the new cases occur in males. Smoking is the most common risk factor for BCa (3). With the rapid increase in tobacco consumption, the rising incidence of bladder cancer poses a significant burden on global healthcare (4). The emergence of immunotherapy has brought new hope for managing BCa patients, with immune checkpoint inhibitors (ICI) being the main treatment modality. They primarily work by inhibiting immune checkpoints to reduce immune suppression and promote anti-tumor immunity. Compared to traditional chemotherapy, ICI therapy offers higher precision and specificity (5).

Ferroptosis is a novel form of programmed cell death, characterized by iron-dependent lipid peroxidation and excessive reactive oxygen species (ROS) accumulation, leading to cell death (6). When intracellular glutathione (GSH) is depleted, glutathione peroxidase 4 (GPX4) becomes inactivated, resulting in the accumulation of lipid peroxides and subsequent cell death (7). Ferroptosis has been associated with the development and progression of multiple types of cancers (8). Some studies have reported that ferroptosis influences cancer development and progression by mediating cancer-associated fibroblasts (9). However, the regulatory mechanisms and targets of ferroptosis in the BCa tumor microenvironment remain unclear.

Single-cell RNA sequencing enables researchers to study tumors with precise details. We can identify novel tumor microenvironment cell subtypes based on single-cell RNA sequencing data and analyze their biological characteristics and prognostic significance. Cell-cell communication analysis reveals important signaling pathways between these cell subtypes and cancer cells, and identifies new targets.

By applying non-negative matrix factorization to single-cell RNA sequencing data, we identify novel ferroptosis-related cell subpopulations within the bladder cancer tumor microenvironment. Combining with classic biological function signatures, we explore the biological characteristics of these cell subpopulations. Cell-cell communication analysis reveals important signaling pathways between these cell subpopulations and cancer cells. Based on bulk RNA-seq data, we evaluate the prognostic significance of these cell subpopulations’ infiltration. Finally, we identify a subtype of cancer-associated fibroblasts (CAFs), ACSL4+CAFs, which impact patients’ overall survival (OS) and sensitivity to immunotherapy. Cell-cell communication analysis reveals SDC1 as an important target on cancer cells interacting with ACSL4+CAFs. Subsequent *in vitro* experiments confirm that SDC1 promotes the proliferation, migration, and invasion of BCa cells.

## Materials and methods

### Acquisition of data

To explore the microenvironment heterogeneity of bladder cancer, single-cell RNA transcriptome data was obtained from the Sequence Read Archive (SRA) (PRJNA662018) (https://www.ncbi.nlm.nih.gov/sra), which contained 8 bladder cancer and 3 normal bladder mucosa tissues (10). To investigate the relationship between ferroptosis and the TME of bladder cancer, we obtained the most frequently studied ferroptosis marker genes from the FerrDb database (http://www.zhounan.org/ferrdb/current/). Besides, six bulk-RNA sequencing datasets were employed to lucubrate the impact of specific ferroptosis subpopulations on patients’ survival, which were obtained from The Cancer Genome Altas (TCGA-BLCA) (https://portal.gdc.cancer.gov/) and the GEO database (GSE48075, GSE32894, GSE31684, GSE160693, GSE13507) (https://www.ncbi.nlm.nih.gov/geo/) (11–15). All the data upon which this study is based are publicly available.

### The processing and visualization of single-cell sequencing data

The CellRanger (v.3.0.1) software was employed to filter and read align the raw single-cell FASTQ data, and feature barcode unique molecular identifier (UMI) matrices were generated based on the human reference genome GRCh38. The Seurat package (v.4.30.1) was used to process the single-cell RNA sequencing expression matrix, cells with gene expression counts less than 200 and cells where mitochondrial gene expression accounted for more than 15% were filtered out. The NormalizeData function was employed to normalize the expression matrix and the RunPCA function was applied to compute the principal components. The UMAP (Uniform Manifold Approximation and Projection) algorithm was utilized to visualize the single-cell RNA sequencing data. Finally, six cell types were identified.

### Identification of the marker genes of ferroptosis-related cell types in TME

The non-negative matrix factorization algorithm was conducted to observe the effect of ferroptosis marker gene expression on TME cell types based on the NMF package (v.0.26). Next, The following criteria were used to determine representative markers for each NMF cell subtype in the FindAllMarkers function: logFC > 0.8, minimum proportion greater than 30%. The cell subpopulations with logFC of ferroptosis marker genes less than 0.5 will be defined as “Non-Ferr,” while those with logFC greater than 0.5 but less than 0.8 will be defined as “Unclear.”

### Function enrichment analysis of ferroptosis-related cell subpopulations

To investigate the biological characteristics of ferroptosis-related cell subpopulations, we performed GO (Gene Ontology) and KEGG (Kyoto Encyclopedia of Genes and Genomes) enrichment analysis based on the clusterProfiler package (v.4.8.3) (16). To explore the metabolic activity of macrophages, we calculated the metabolism enrichment scores based on the scMetabolism package (v.0.2.1). Besides, the AUCell package (v.1.22.0) was utilized to quantify the biology activities.

### SCENIC analysis for ferroptosis-related cell subpopulations

To clarify the gene regulatory network of transcription factors (TFs) in TME cell subpopulations, the SCENIC package (v.1.3.1) was employed. Two gene-motif rankings (hg19-500bp-upstream-7species.mc9nr.feather and hg19-tss-centered-10kb-7species.mc9nr.feather) were downloaded from the RcisTarget database (https://github.com/aertslab/RcisTarget) to identify the transcription start site (TSS). Then, potential TF-target relationships were recognized and a co-expression gene network was constructed. Only TFs with False Discovery Rate (FDR) <0.05 were considered in this study.

### Cell-cell communication analysis for ferroptosis-related cell subpopulations

The CellChat package (v.1.6.1) was utilized to construct the intratumor communications networks. CellChatDB.human was employed to evaluate the signaling pathway inputs and outputs between TME cell subpopulations and cancer cells. Next, the computeCommunProPathway and aggregateNet functions were used to calculate the cell-cell communication network and communication strength. Finally, the netVisual_bubble function was performed to visualize ligand-receptor interactions based on the human ligand-receptor pairs database.

### Pseudotime trajectory analysis for ferroptosis-related cell subpopulations

To explore the role of the ferroptosis marker genes in the trajectory of cellular development and differentiation, we employed pseudotime trajectory analysis for TME cell subpopulations based on the Monocle package (v.2.22.0). Highly variable genes were filtered according to the following criteria: mean_expression ≥ 0.1 and dispersion_empirical ≥ 1*dispersion_fit. The method for dimensionality reduction was DDRTree. Next, the plot_pseudotime_heatmap function was employed to show the pseudotime heatmap, and the plot_cell_trajectory function was used to illustrate the dynamic expression of ferroptosis marker genes in TME.

### Assessment of immune infiltration and ICI therapy

Four algorithms were utilized to compare immune cell infiltration across different groups, including CIBERSORT, XCell, EPIC, and Quantiseq. Subsequently, the ESTIMATE package (v.1.0.13) was utilized to calculate the abundance of TME components. The Tracking Tumor Immunophenotype (TIP) algorithm (http://biocc.hrbmu.edu.cn/TIP/) was employed to assess the cancer immunity cycle. The online website Tumor Immune Dysfunction and Exclusion (TIDE) (http://tide.dfci.harvard.edu/login/) was utilized to assess the ICI response of bladder cancer patients, as well as the Subclass Mapping (Submap) algorithm.

### Survival analysis for specific TME cell subpopulations

The ssgsea function was employed to calculate the infiltration levels of specific cell subtypes in bladder cancer patients based on the GSVA package (v.1.42.0). To investigate the prognostic significance of specific TME cell subpopulations, the survival (v.3.2.13) and survminer (v.0.4.9) package were employed to conduct the survival analysis. Patients were divided into two groups according to the optimal cutoff. All survival analyses in this study were subjected to log-rank tests.

### Cell culture, transfection, and interference

The study utilized human bladder cancer cells (UM-UC-3 and T24) from the Cell Bank of the Chinese Academy of Sciences (Shanghai, China). UM-UC-3 was cultured in high glucose DMEM and T24 was cultured in RPMI-1640 with 10% fetal bovine serum (Gibco, USA) and 1% streptomycin/penicillin (Thermo Fisher Scientific, USA) in an incubator at 37°C and 5% CO2. The cells were transferred upon reaching a cell density of 70-80%, and the medium was changed daily.

The siRNA targeting SDC1 (siSDC1) lentivirus was purchased from GeneChem (shanghai, China) to suppress the SDC1 gene of T24 and UM-UC-3 cells. The siSDC1 was transfected into T24 and UM-UC-3 cells with polyethylene, then the cells were screened with puromycin. The siRNA sequences can be obtained in **Supplementary Table 1**.

### Colony formation assay

UM-UC-3 and T24 cells were seeded onto 6-well plates at a density of 1000 cells per well and allowed to culture for 2 weeks until the formation of cell colonies. Subsequently, the cells were washed three times with phosphate-buffered solution (PBS) (Yeasen, China), fixed with 4% methanol for 15 minutes, stained with 0.5% crystal violet solution for 30 minutes, and analyzed using ImageJ software.

### Wound-healing assay

To analyze cell direct migration, a wound-healing assay was conducted. UM-UC-3 and T24 cells were inoculated in a 6-well plate and cultured until reaching 70%-80% density. The cell monolayer was gently scratched using the tip of a sterile 200µL pipette after removing the medium. Subsequently, the wells were rinsed twice with PBS, and serum-free medium was added for continued culture. Images were captured at 0-, 12-, and 24-hours post-scratching and analyzed using ImageJ software.

### Cell viability detection

A CCK-8 assay kit (Biosharp, China) was used to assess cell viability. After the intervention, cells were seeded on 96-well plates and incubated at 37°C with 5% CO2. UM-UC-3 and T24 cells were treated with a diluted CCK-8 solution for 2h. The absorbance values at 450nm were quantified using a microplate reader. (Thermo Fisher Scientific, USA).

### EdU assay

Following the manufacturer’s instructions, the EdU detection assay (Beyotime, China) was used to detect different cell states after a series of operations, DNA synthesis, and cell proliferation was observed through fluorescence microscopy (Olympus, USA).

### Flow cytometric analysis

The Annexin V-FITC/PI Apoptosis (Beyotime, China) kit was used to detect the apoptosis rate of BCa cells, including early and terminal apoptosis. BD flow cytometry (BD FACSLyric, USA) was employed to analyze cell samples.

### Transwell migration and invasion assay

The transwell assays were performed to observe the migration and invasion ability. T24 and UM-UC-3 cells were seeded at a density of 2×10^4^ cells per well in the upper chambers, with 200µL serum-free medium, while the lower chambers contained 800µL of medium supplemented with 10% serum. To perform the invasion assay, 50mg/L Matrigel glue was covered in the upper chambers. After placing the transwell chambers (Corning, USA) in a 37°C, 5% CO2 incubator for 48 hours, 4% methanol was used to fix cells for 30 minutes, and 0.5% crystal violet was employed to stain for 30 minutes. Finally, the results can be obtained by taking photographs and counting.

### Statistical analysis

All data processing and statistical analysis performed in this study were based on R software (v.4.3.1). To verify the differences among various groups, diverse tests (Wilcoxon rank-sum test, Fisher exact test, Student’s t-test, Kruskal-Wallis test) were performed. In correlation analysis, the Pearson test was used to verify the statistical significance. All experiment data were presented as the mean ± SD, and GraphPad Prism 8 software was employed to analyze these experiment data. In this study, only a two-sided p-value below 0.05 was considered statistically significant.

## Results

### The landscape of ferroptosis-related genes in TME of BCa

**Figure 1:**
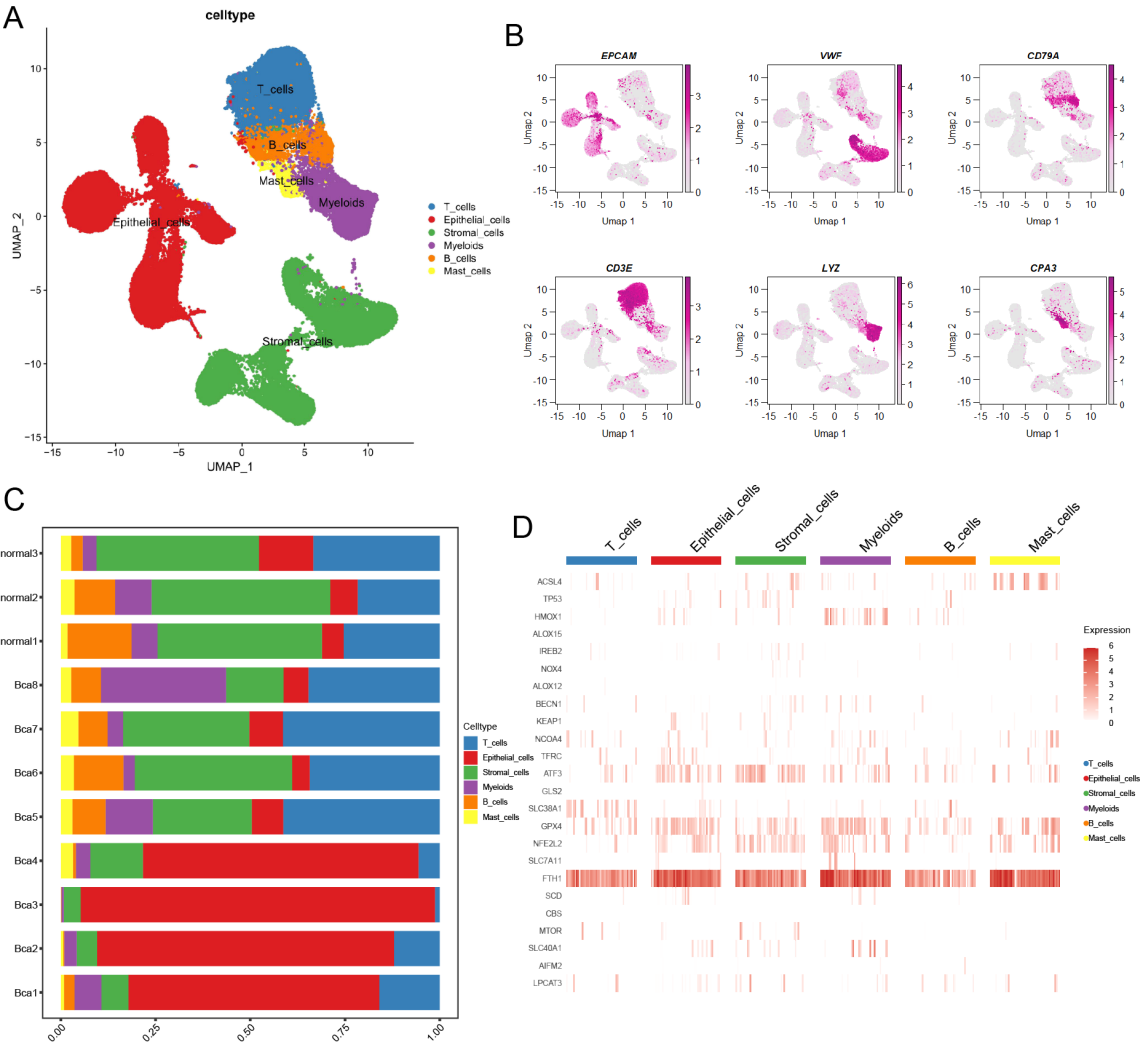
Overview of ferroptosis-related marker genes in scRNA transcriptome data of BCa. **(A)** The landscape of main cell types was illustrated by UMAP. **(B)** The heatmap showed the classical marker genes in the BCa landscape. **(C)** A bar chart illustrated the proportion of different cell types across various samples. **(D)** The heatmap displayed the distribution of ferroptosis-related marker genes in main cell types.

A total of 83,146 cells were mapped onto the cell atlas and annotated into six major cell types (**Figure 1A**). Subsequently, the classical cell markers were displayed in the cell atlas according to their expression levels (**Figure 1B**). Additionally, the cell atlas comprised tissue samples from a total of 11 patients, including three normal tissues and eight cancer tissues, enabling observation and comparison of the cellular composition proportions across different samples (**Figure 1C**). Furthermore, the differential expression of ferroptosis-related genes across different cell types can be observed through the heatmap (**Figure 1D**), as a ferroptosis suppressor gene, GPX4 had higher expression in epithelial cells and myeloid cells across major cell types, which meant these two cell types had lower ferroptosis level.

### Novel ferroptosis-related CAFs mediated TME of BCa

**Figure 2:**
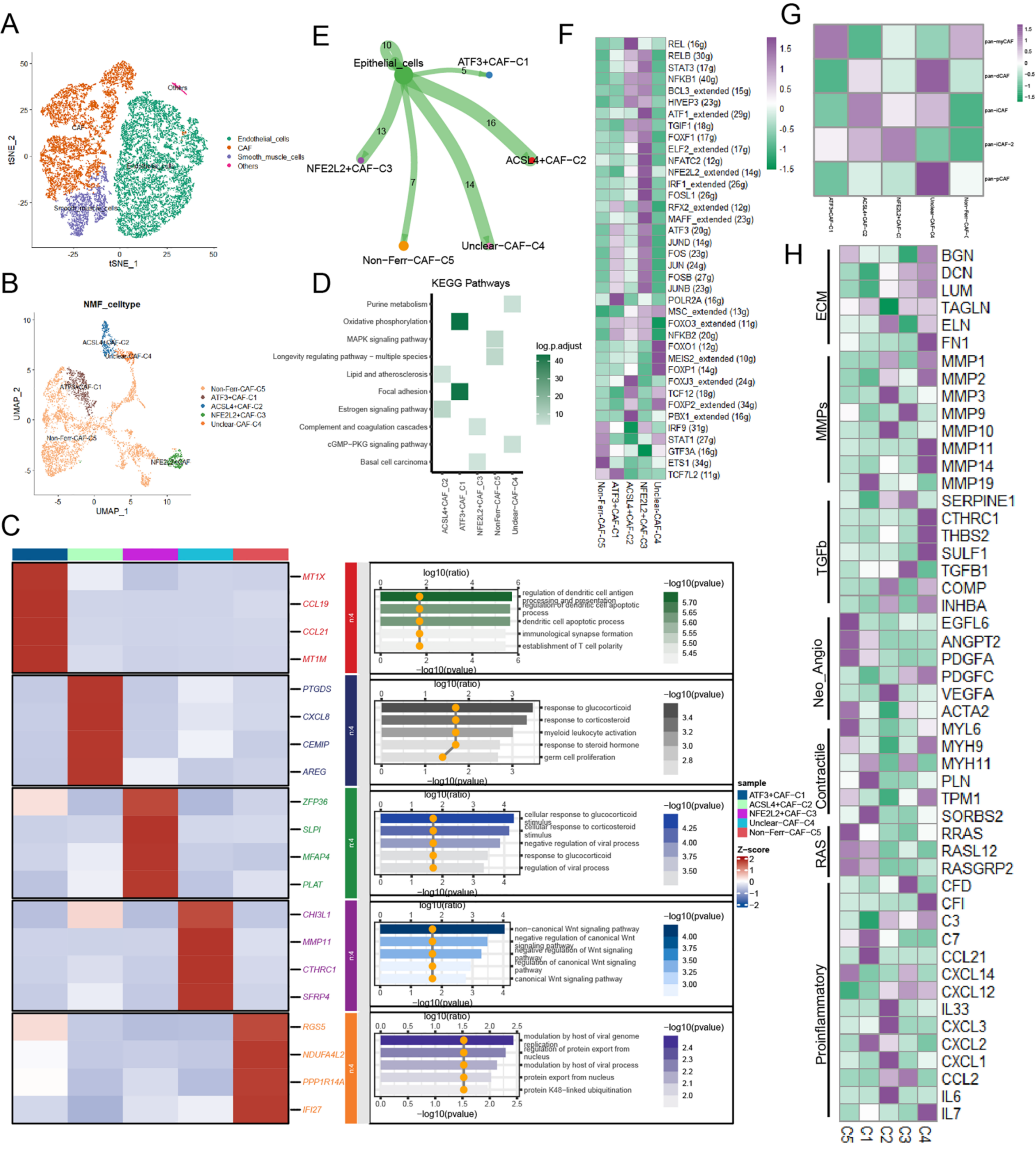
The landscape of ferroptosis-related marker genes in CAFs. **(A)** Presence of CAFs in stromal cells. **(B)** The ferroptosis-related CAF subpopulations were illustrated by UMAP. **(C)** The heatmap displayed the differential genes and biological functions of each subpopulation. **(D)** Activation of the KEGG pathway of each subpopulation. **(E)** The cell-cell communication strength between the ferroptosis-related CAF subpopulations and cancer cells. **(F)** Transcriptional regulatory factors for each cell subpopulation. **(G)** Correlations between the ferroptosis-related CAF subpopulations and classical CAF signatures (p < 0.05). **(H)** Heatmap illustrated the distinct average expression levels of prevalent signaling pathway genes among the five subpopulations of CAFs associated with ferroptosis, encompassing Proinflammatory, RAS, Contractile, Neo-Angio, TGFb, MMPs, and ECM.

According to classical marker genes, stromal cells were annotated into 3 major cell types: endothelial cells, smooth muscle cells, and CAFs (**Figure 2A**; **Supplementary Figure 1A**). In this section, we primarily focused on CAFs. After undergoing NMF dimensionality reduction and clustering, CAFs were subdivided into 5 novel cell subpopulations (**Figure 2B**). We found that ACSL4+CAFs interacted with glucocorticoid activities and myeloid leukocyte activation (**Figure 2C**). KEGG analysis yielded that ACSL4+CAFs were associated with estrogen signaling pathways, and ATF+CAFs exhibited active oxidative phosphorylation (**Figure 2D**). To explore the relationship between CAF subpopulations and cancer cells, cell-cell communication analysis yielded that ACSL4+CAFs had the strongest communication with cancer cells among these novel ferroptosis-related CAFs (**Figure 2E**). Furthermore, ligands-receptors analysis uncovered that ACSL4+CAFs were strongly associated with the EGF (Epidermal Growth Factor) and MIF (Macrophage migration Inhibitory Factor) signaling pathway (**Supplementary Figures 1B, C**).

TFs of these novel ferroptosis-related CAFs were illustrated in the gene regulatory network based on the SCENIC analysis, and FOXJ3, TCF12, FOXP2, and PBX1 were upregulated in ACSL4+CAFs (**Figure 2F**). Next, to further investigate the biology characteristics, we assessed the correlation among Pan-CAF signatures and found that ACSL4+CAFs were similar to inflammatory CAF (pan-iCAF) (**Figure 2G**). Then, the heatmap highlighted the same conclusion: ACSL4+CAFs were closely related to inflammation within BCa (**Figure 2H**). Pseudotime trajectory analysis exhibited the development of CAFs and dynamic expression of marker genes, the heatmap reflected ferroptosis-related genes played important roles in CAFs within BCa (**Supplementary Figure 1D**), we also found that ACSL4+CAFs were at the beginning of trajectory and ATF+CAFs were at the end of trajectory (**Supplementary Figures 1E, F**).

### Ferroptosis-related macrophages exhibit particular biological features

**Figure 3:**
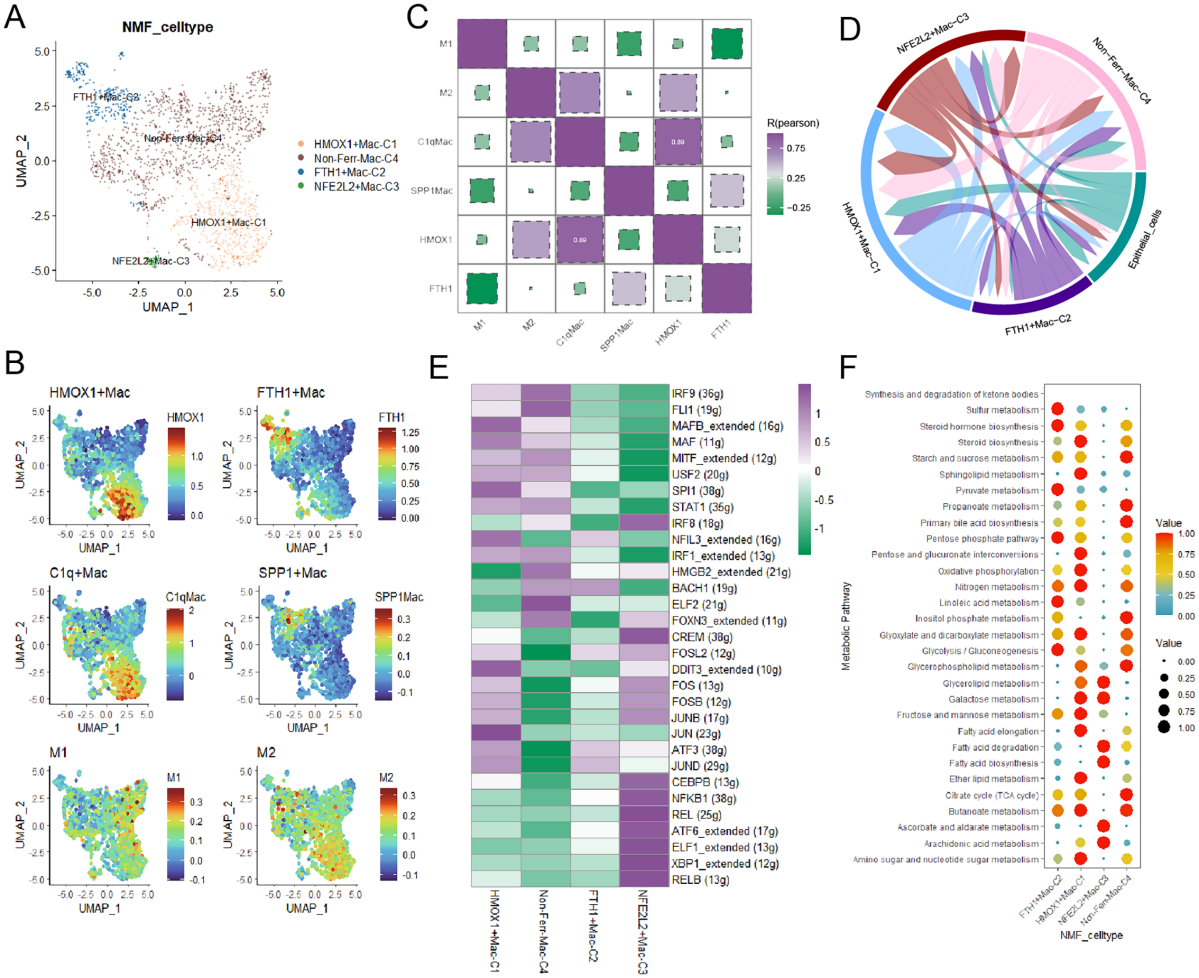
The landscape of ferroptosis-related marker genes in macrophages. **(A)** The ferroptosis-related macrophage subpopulations were illustrated by UMAP. **(B)** The heatmap demonstrated the distinct biological features in macrophages. **(C)** Correlations between the ferroptosis-related macrophage subpopulations and classical macrophage signatures. **(D)** The circular plot visualized the strength of cell-cell communications between macrophages and cancer cells. **(E)** Transcriptional regulatory factors for each cell subpopulation. **(F)** The metabolism landscape of ferroptosis-related macrophage subpopulations.

Macrophages played a crucial role in TME in the progress of BCa, unique macrophage subpopulations exhibited distinct biological features. Firstly, myeloid cells were annotated into three major cell types based on classical marker genes (**Supplementary Figures 2A, B**). We identified three novel macrophage subpopulations through NMF dimensionality reduction (**Figure 3A**). By integrating specific macrophages-related signatures reported in previous literature, we gained a deeper understanding of the biological characteristics of these macrophage subpopulations and their potential roles in BCa, HMOX1+macrophages shared biological similarities with C1q+ macrophages and M2 macrophages, and FTH1+macrophages were closely related to SPP1+macrophages (**Figures 3B, C**). Then, pseudotime trajectory analysis yielded that these ferroptosis-related marker genes, especially FTH1, played a crucial role in the development of macrophages (**Supplementary Figures 2C, D**). Subsequently, cell-cell communication analysis indicated that HMOX1+macrophages had the most interactions with cancer cells, while FTH1+macrophages had the fewest (**Figure 3D**, **Supplementary Figure 2E**). Signaling pathway analysis uncovered that the SPP1 signaling pathway was upregulated in FTH1+macrophages, and IL6 and IL10 were overexpressed in HMOX1+macrophages (**Supplementary Figures 2F, G**). Next, the regulatory network illustrated some TFs, such as JUN, JUNB, and ATF3, were upregulated in HMOX1+macrophages (**Figure 3E**).

To deeply understand the biology characteristics of these macrophage subpopulations, we performed GO enrichment analysis, and the results yielded that HMOX1+macrophages were associated with protein refolding, those genes upregulated in FTH1+macrophages were enriched in chemokine-mediated signaling pathway and cellular response to chemokine (**Supplementary Figure 2H**). Finally, the scMetabolism package showed that FTH1+macrophages had active sulfur and pyruvate metabolism, and HMOX1+macrophages exhibited vigorous steroid biosynthesis and oxidative phosphorylation (**Figure 3F**).

### The landscape of ferroptosis-related T cells in TME

**Figure 4:**
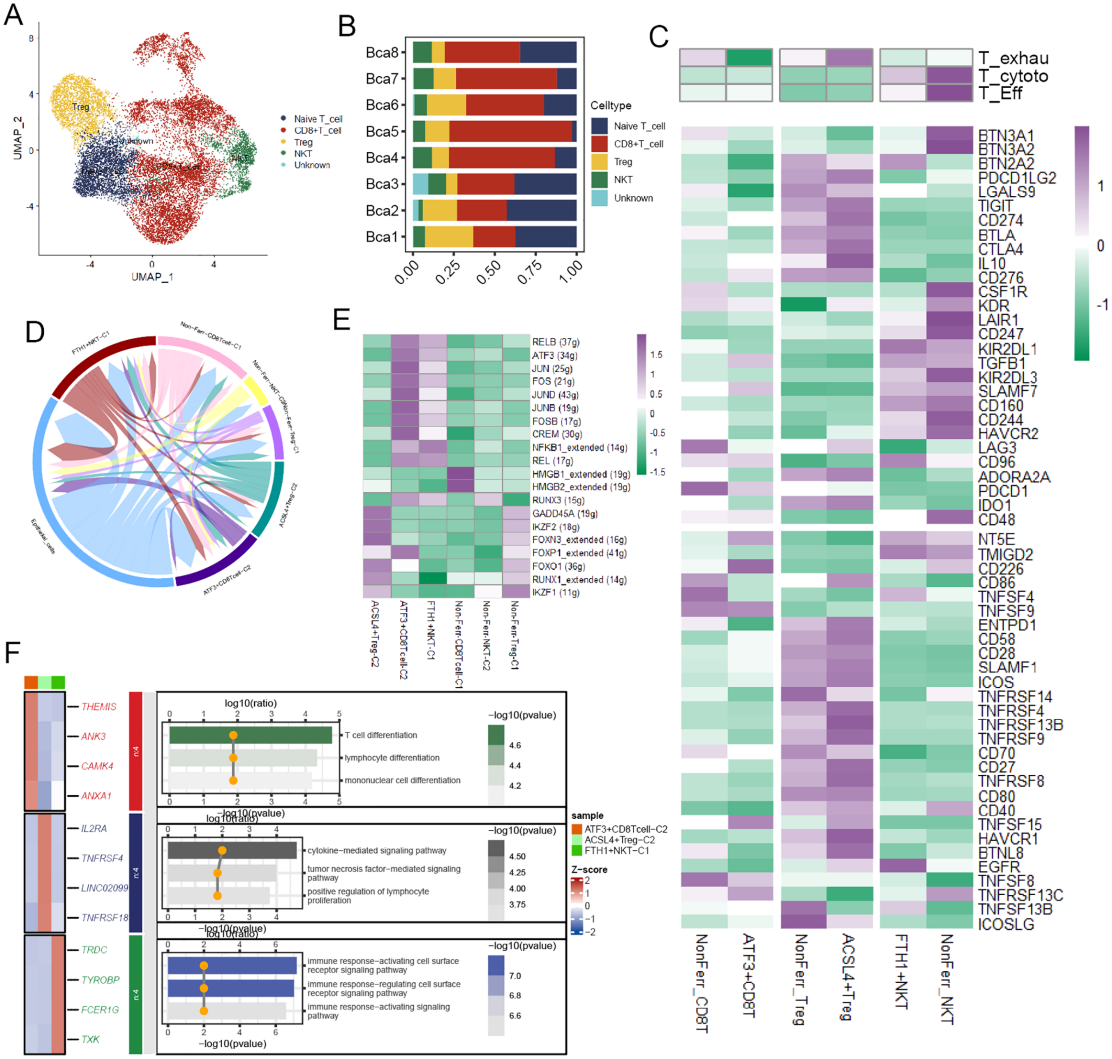
The biological features of the ferroptosis-related T cell subpopulations. **(A)** The landscape of T cells in BCa. **(B)** A bar chart illustrated the proportion of different T cell types across various samples. **(C)** The heatmap depicted the markedly distinct characteristics across ferroptosis-related subpopulations of T cells, encompassing CD8+T cells, NKTs, and Tregs. These features include T exhaustion score, T cytotoxic score, T effector score, various immune co-stimulators, and co-inhibitors (Kruskal-Wallis test, p < 0.001). **(D)** The circular plot visualized the strength of cell-cell communications between T cells and cancer cells. **(E)** Transcriptional regulatory factors for each cell subpopulation. **(F)** The heatmap displayed the differential genes and immune pathways of each subpopulation.

T cells were divided into four cell types according to their distinct gene expression (**Figure 4A**), and the bar plot illustrated their proportions in BCa patient samples (**Figure 4B**). These T cell types were annotated into six cell subpopulations after undergoing NMF dimensionality reduction. To deeply unravel the molecular characteristics, we calculated some T cell-related signature scores in these cell subpopulations. The result demonstrated that compared to ATF3+CD8T cells, NonFerr_CD8T cells exhibited higher T cell exhaustion scores, while ACSL4+Tregs had higher scores than NonFerr_Tregs. Interestingly, compared to FTH1+NKT (Natural Killer T cells), NonFerr_NKT were biologically analogous to effector T cells and cytotoxic T cells (**Figure 4C**). Subsequently, cell-cell communication analysis unraveled that ATF3+CD8T cells and FTH1+NKT occupied most interactions with the cancer cells (**Figure 4D**). Next, we observed that some TFs, such as IKZF1, RUNX1, FOXO1, FOXP1, and FOXN3, were upregulated in ACSL4+Tregs, and in ATF3+CD8T cells, JUN, FOS, JUND, JUNB, FOSB were overexpressed (**Figure 4E**). Finally, enrichment analysis yielded that ATF3+CD8T cells were associated with lymphocyte differentiation, ACSL4+Tregs were associated with tumor necrosis factor and cytokine regulation pathways, and FTH1+NKT were associated with cell surface receptor signaling pathways (**Figure 4F**).

### Prognostic significance of specific ferroptosis-related TME cell subpopulations

**Figure 5:**
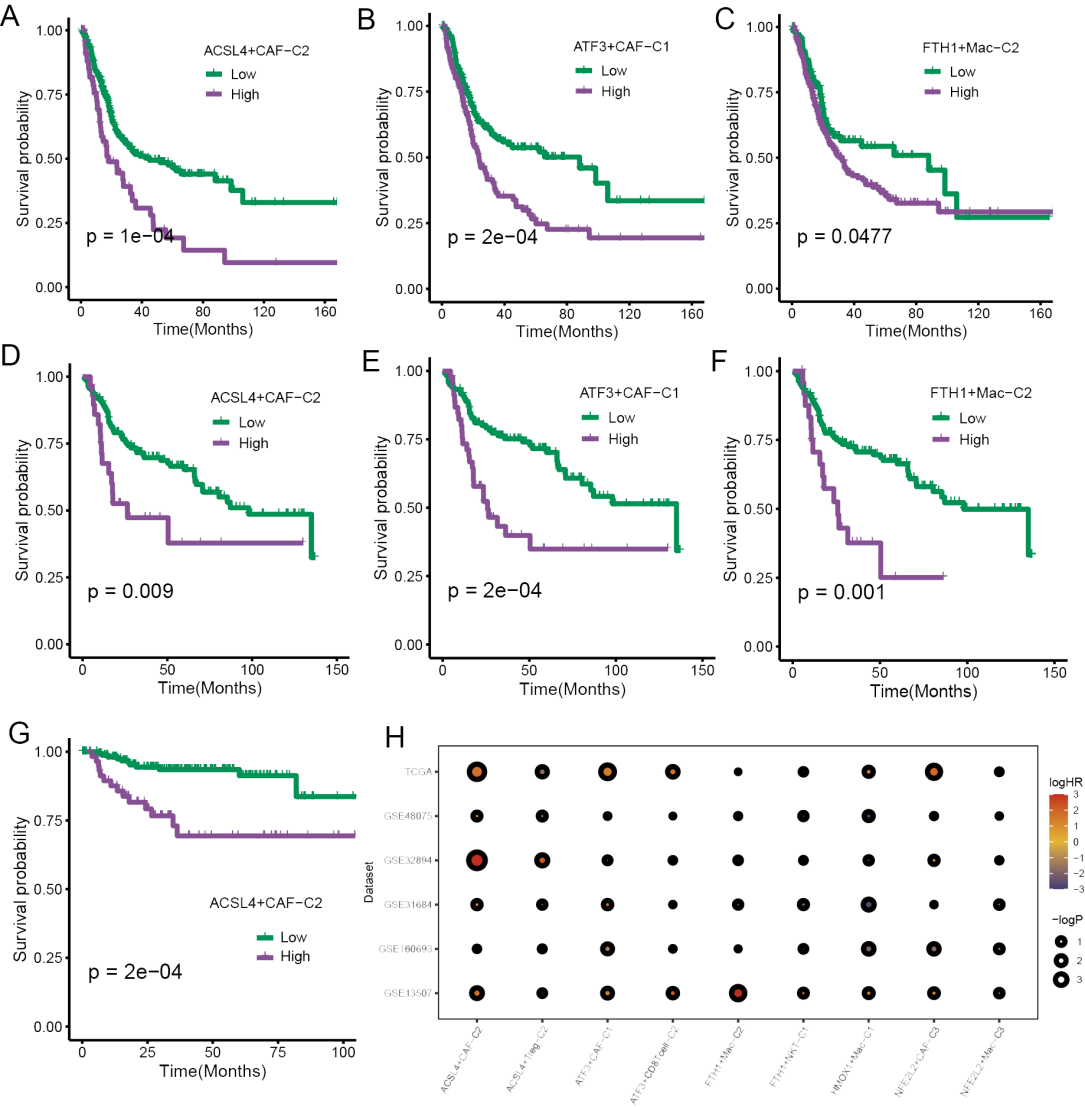
The prognostic significance of specific ferroptosis-related TME cell subtypes. **(A–C)** The K-M curve plots demonstrated the impact of specific TME cell infiltration on OS in the TCGA cohort. **(D–F)** The K-M curve plots demonstrated the impact of specific TME cell infiltration on OS in the GSE13507 cohort. **(G)** The K-M curve plots demonstrated the impact of specific TME cell infiltration on OS in the GSE32894 cohort. **(H)** The bubble heatmap illustrated the prognostic significance in univariate Cox regression analysis.

The FindAllMarkers function was employed to calculate differential expression genes (DEGs) of those cell subpopulations, and we extracted the top 50 for each subpopulation to obtain the signatures of specific ferroptosis-related TME cell subpopulations. The survival analysis was performed to validate the prognostic significance of the signatures of those subpopulations, and we found that a few TME cell subpopulations were closely associated with overall survival (OS) in the TCGA cohort, including CAFs and macrophages (**Figures 5A–C**). Then, the same results were validated again in the GSE13507 cohort (**Figures 5D–F**). subsequently, the survival analysis performed in the GSE32894 yielded that only ACSL4+CAFs related to OS (**Figure 5G**).

To ensure the rigor of the results, Cox regression analysis was implemented using six BCa cohorts. The results yielded a high infiltration of ACSL4+CAFs was closely associated with adverse effects on patients’ OS (**Figure 5H**).

### ACSL4+CAFs impacted immune infiltration and ICI therapy

**Figure 6:**
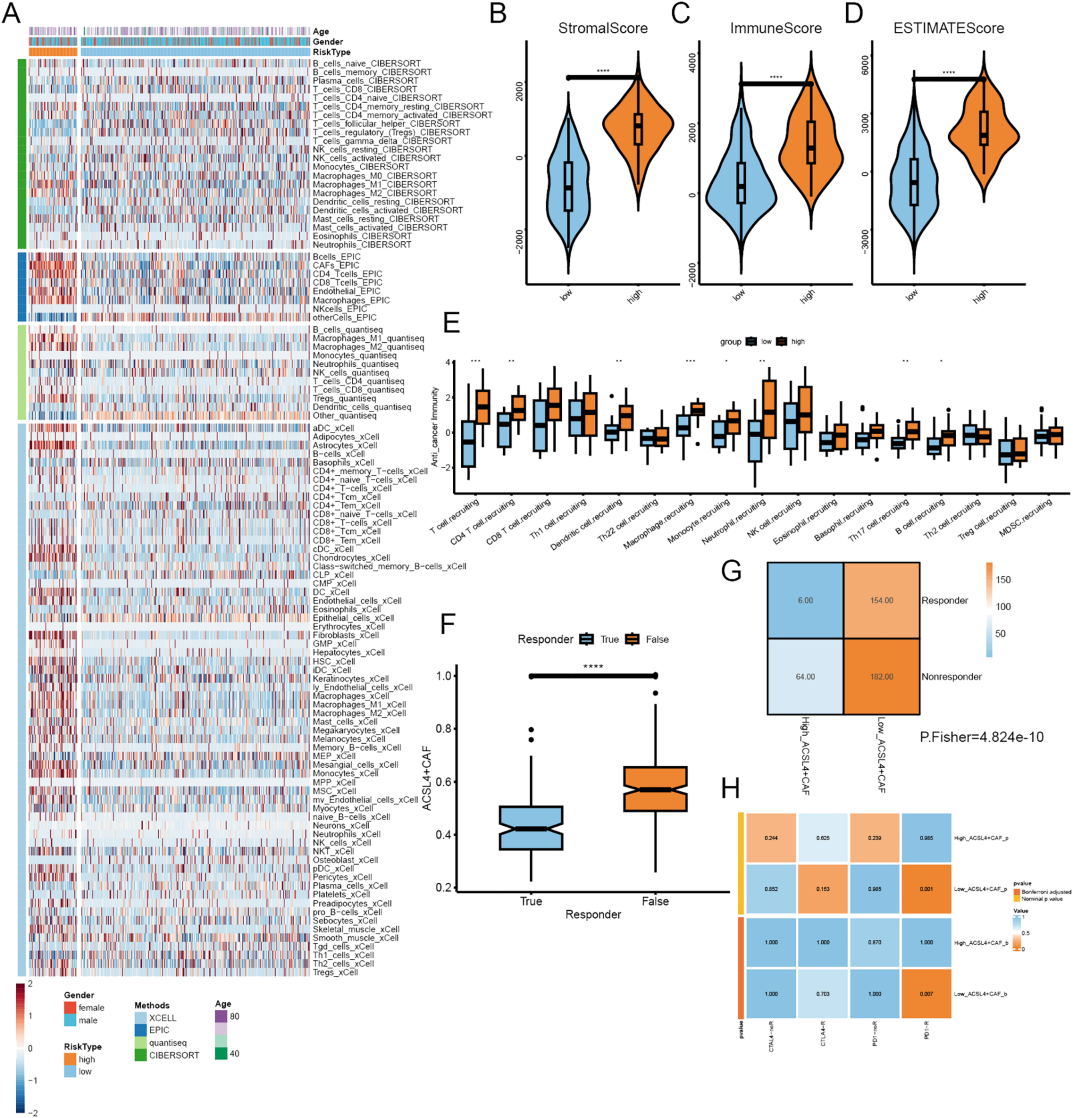
The immune infiltration of different ACSL4+CAFs groups. **(A)** The landscape of immune cells infiltration of TCGA cohort. **(B-D)** The distribution of immune components between the two groups. **(E)** Differences in the anti-tumor immunity cycle between the two groups. **(F)** Box plot showed the different ACSL4+CAFs infiltration levels between distinct immunotherapy responses in the TCGA cohort. **(G)** Contingency table between immunotherapy responses and ACSL4+CAFs infiltration based on TIDE in the TCGA cohort. **(H)** The contingency table presented the relationship between ACSL4+CAFs infiltration and optimal immunotherapy (anti-PD1, anti-CTLA4). *P < 0.05, **P < 0.01, ***P < 0.001, ****P < 0.0001.

To explore the impact of ACSL4+CAFs on immune infiltration and response to ICI, we calculated immune infiltration in the TCGA cohort and evaluated ICI response. All patients were divided into two groups based on the optimal cutoff; a total of four algorithms were employed to assess the immune cell infiltration in BCa, and the heatmap illustrated a remarkable difference between the two groups, especially macrophages, Tregs and NKT (**Figure 6A**). the ESTIMATE algorithm was implemented to calculate the abundance of TME components, we found that high ACSL4+CAFs group had more complex TME than low ACSL4+CAFs group (**Figures 6B–D**). To further explore the difference between the two groups in the immunity cycle, the TIP algorithm yielded that the high ACSL4+CAFs group exhibited a stronger capacity for immune cell recruitment (**Figure 6E**). To further investigate the difference between the two groups in ICI therapy response, the TIDE algorithm revealed that patients who did not respond to ICI therapy had higher ACSL4+CAFs infiltration (**Figure 6F**), similarly, there was a higher proportion of non-responsive patients to ICI treatment within high ACSL4+CAFs group (**Figure 6G**). Next, we discovered that the low ACSL4+CAFs group appeared to be more sensitive to anti-PD-1 treatment (**Figure 6H**).

### The close association between ACSL4+CAFs and BCa cells

**Figure 7:**
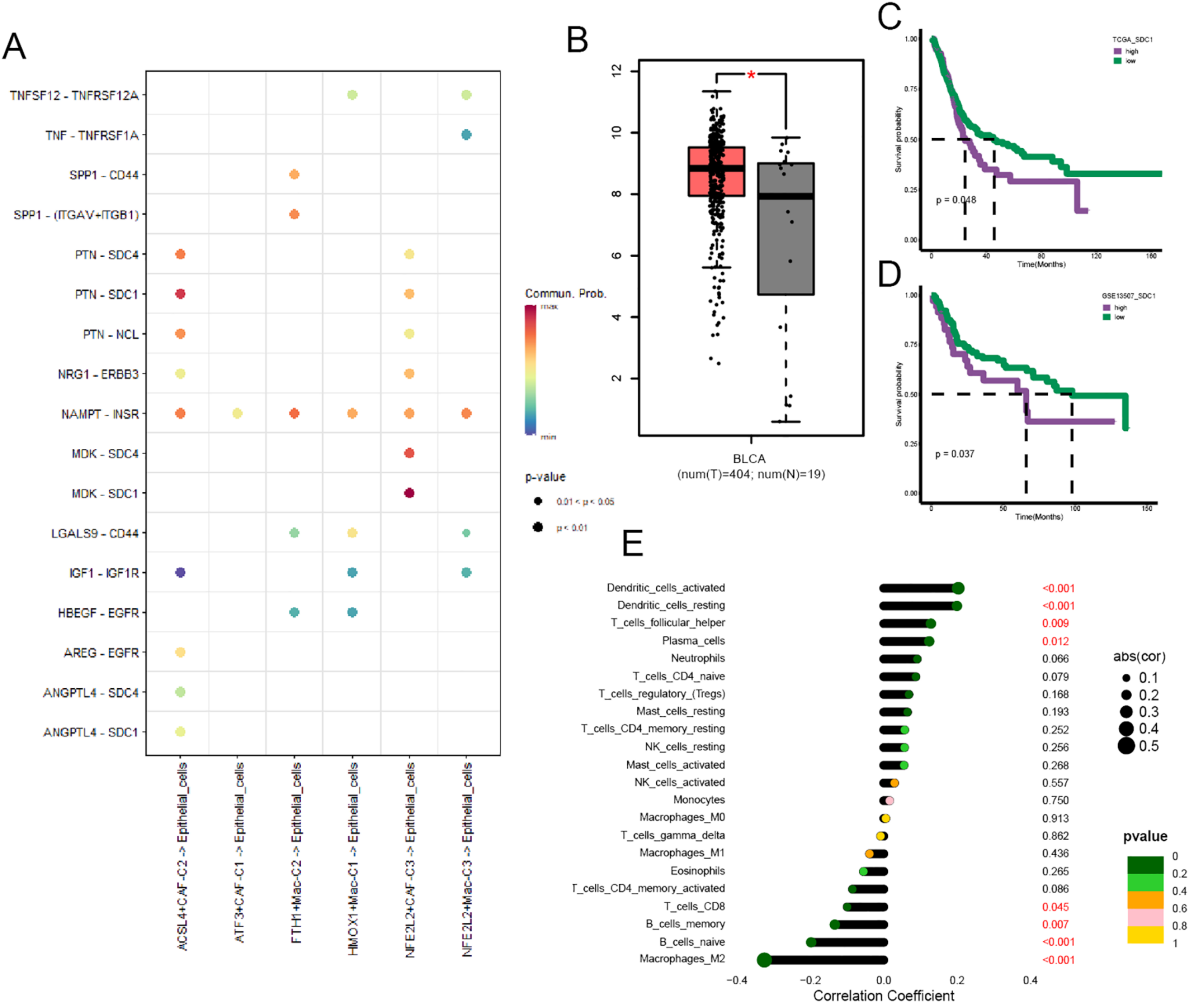
The prognostic significance of SDC1 in BCa. **(A)** The dot plot illustrated the communications between those specific TME cell subpopulations and cancer cells. **(B)** Box plot displaying the different expressions of SDC1 between tumor and normal tissue. **(C, D)** The K-M curve plot detected the prognostic significance of SDC1 among the TCGA and GSE13507 cohort. **(E)** The correlation between SDC1 and immune cell infiltration in BCa. *P < 0.05.

We performed cell-cell communication analysis between cancer cells and a few specific ferroptosis-related TME cell subpopulations, and the results yielded that SDC1 was a crucial target associated with ACSL4+CAFs (**Figure 7A**). The GEPIA website showed that BCa tissues had a higher SDC1 expression than normal tissues (**Figure 7B**), and we performed survival analysis within two cohorts to investigate the prognostic significance of SDC1, which revealed that higher SDC1 expression had an adverse impact on patients’ OS (**Figures 7C, D**). Subsequently, the correlation analysis showed that SDC1 negatively correlated to CD8+ T cells in BCa (**Figure 7E**).

### SDC1 promoted the proliferation and invasion ability of BCa cells

**Figure 8:**
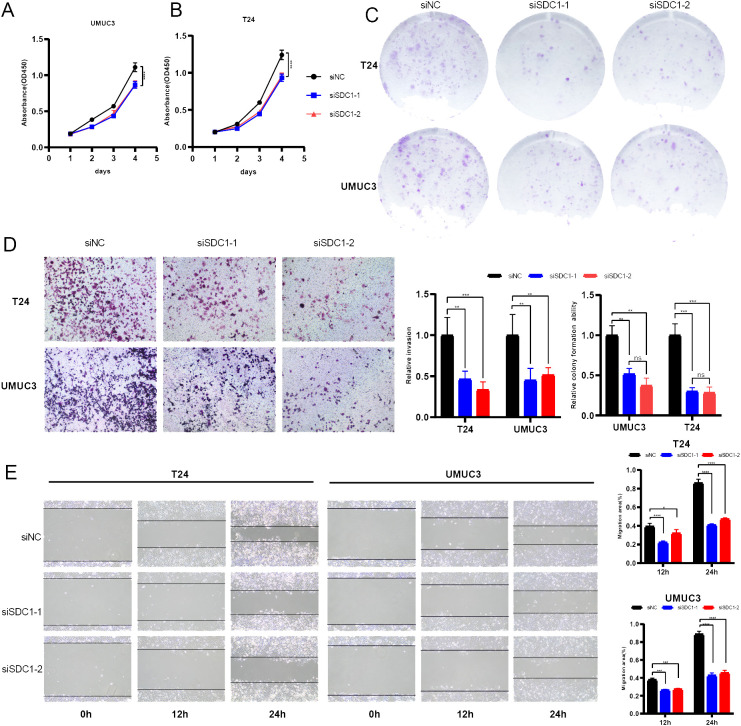
*In vitro* experiments. **(A, B)** Line plots showed that SDC1 enhanced the viability of BCa cells. **(C)** Colony formation assays displayed that SDC1 increased the colony numbers. **(D)** Transwell assays showed knocking down SDC1 inhibited the invasion ability of BCa cells. **(E)** Wound-healing assays showed that SDC1 promoted the direct migration of BCa cells. *P < 0.05, **P < 0.01, ***P < 0.001, ****P < 0.0001. ns: no significance.

A series of *in vitro* experiments were performed to verify the function of SDC1 in BCa cells. CCK-8 assays yielded that SDC1 enhanced the viability of BCa cells (**Figures 8A, B**), and the colony formation assays showed that SDC1 remarkably increased the number of colonies (**Figure 8C**). To assess the different invasion abilities of BCa cells, the transwell assays were performed to display that the invasion ability was decreased after SDC1 was knocked down (**Figure 8D**). The wound-healing assays illustrated that SDC1 promoted the direct migration of BCa cells (**Figure 8E**).

**Figure 9:**
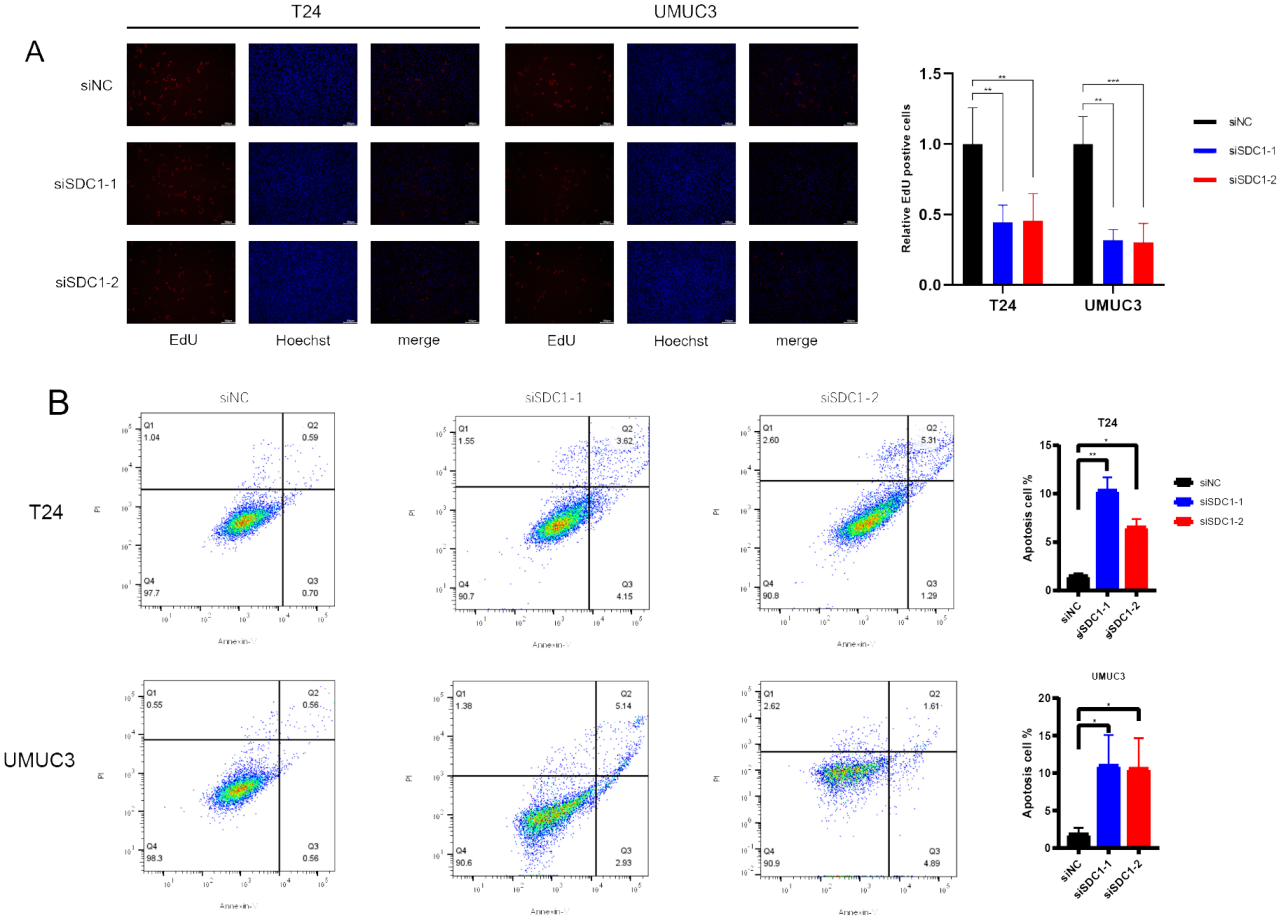
*In vitro* experiments. **(A)** EdU assays showed that SDC1 enhanced cell proliferation. **(B)** Flow cytometric analysis yielded that SDC1 suppressed apoptosis in BCa cells. *P < 0.05, **P < 0.01, ***P < 0.001.

The EdU assay was employed to quantify the proliferation level of cells, and the results indicated that knocking down SDC1 significantly inhibited the proliferation capability of BCa cells (**Figure 9A**). Furthermore, flow cytometric analysis revealed that knocking down SDC1 led to a significant increase in the number of apoptotic BCa cells, suggesting that SDC1 plays a role in inhibiting apoptosis in BCa cells (**Figure 9B**).

## Discussion

Ferroptosis, as a novel form of cell death, has played a significant role in various cancers (17). Lipid peroxidation (LPO) and increased iron load have served as important signals for ferroptosis (18). Recent research suggests that ferroptosis could be a crucial target for cancer therapy (19). Apatinib, a tyrosine kinase inhibitor, has been reported to induce ferroptosis in gastric cancer cells by inhibiting GPX4 activity (20). P53 mutations, common events in tumor development, have been shown to inhibit ferroptosis and promote tumor progression (21). Additionally, the direct use of ferroptosis inducers as chemotherapy drugs holds promise. Erastin, a typical inducer of ferroptosis, can reduce the cellular synthesis of GSH by inhibiting SLC7A11, leading to increased LPO and subsequent ferroptosis (22). Some studies indicate that the anti-tumor efficacy of combined Erastin and platinum drugs is stronger than that of platinum drugs alone (23). Therefore, ferroptosis may offer a new therapeutic strategy for treating chemotherapy-resistant patients (24).

Some studies have indicated that when levels of free iron increase in bladder cancer cells, cell proliferation is inhibited, suggesting a close association between ferroptosis and bladder cancer cell proliferation (25). Recent studies have developed a novel targeted therapy approach for bladder cancer. CPNPs, a type of conjugated polymer nanoparticle carrying iron ions, can induce ferroptosis in cancer cells by releasing iron ions upon entry into tumor cells (26). It has been reported that CPNPs can kill 80% of cancer cells under high-dose conditions. These findings offer promising management strategies for patients with BCa.

TME, as a crucial component of bladder cancer, plays a significant role in tumor progression (27). CD8+ T cells, as the primary mediators of anti-tumor immunity, are also affected by ferroptosis (28). Literature suggests that tumor-derived CD8+ T cells accumulate more LPO compared to lymph node-derived CD8+ T cells (29). Furthermore, studies have reported that overexpression of GPX4 in CD8+ T cells can protect them from the effects of ferroptosis, restore their secretion of cytotoxic factors, and increase the infiltration of CD8+ T cells within tumors (30). Tregs, classical immunosuppressive components in the TME, exhibit significantly lower levels of LPO than CD8+ T cells (31). Moreover, activation of TCR/CD28 in Tregs induces GPX4 expression, thereby inhibiting ferroptosis (32). These findings suggest that Tregs rarely undergo ferroptosis. Macrophages in bladder cancer are generally classified as anti-tumor M1 subtype or pro-tumor M2 subtype (33). Some iron-targeting nanoparticles have been developed to repolarize M2 macrophages into M1 subtype, assisting in tumor treatment (34). Recent studies indicate that tumor-derived NK cells express increased levels of proteins associated with ferroptosis and lipid peroxidation (35), with their mitochondria resembling those of ferroptotic cells. LPO leads to a metabolic imbalance in NK cells, causing functional impairment (36). In conclusion, ferroptosis is closely related to the bladder cancer TME.

Ferroptosis is closely linked to immunotherapy as well (37). Studies have found that cancer cells undergoing ferroptosis exhibit dual characteristics (38). On one hand, ferroptotic cancer cells release immunostimulatory signals, attracting macrophages, dendritic cells, and other immune cells to the tumor site (39). The enhanced immunogenicity also induces tumor-specific immune responses (40). On the other hand, it has been reported that ferroptotic cancer cells can release 8-hydroxy-2′-deoxyguanosine (8-OHdG), which promotes M2 polarization (41). These products originate from oxidative DNA damage (42). Cytotoxic CD8+ T cells secrete interferonγ (IFNγ), which inhibits SLC7A11 by activating the JAK/STAT1 pathway in cancer cells, thereby inducing ferroptosis in cancer cells (43). This reveals a new mechanism of anti-tumor immunity. However, cancer cells undergoing ferroptosis also release immunosuppressive signals, promoting the infiltration of immunosuppressive cells, and leading to feedback protection (44). In summary, there is a complex crosstalk between ferroptotic cancer cells and immune cells during anti-tumor immune processes (45). Some studies have reported that ferroptosis inducers can significantly enhance the efficacy of ICI therapy (46, 47). However, due to the diversity of ferroptosis pathways, the application of a single inducer in multiple cancers may not be practical. Therefore, selecting specific inducers for combination with ICI is worth considering (48).

In this study, utilizing NMF dimensionality reduction clustering, we identified a novel subpopulation of ACSL4+CAFs in the BCa TME. Subsequent survival analysis and immune infiltration assessment revealed that high infiltration of this subpopulation indicates poor prognosis and lack of response to ICI. Enrichment analysis of this subpopulation revealed its association with estrogen response, which was noteworthy given the clinical characteristic of poorer prognosis in female BCa patients compared to males (49). Furthermore, we observed frequent crosstalk between ACSL4+CAFs and cancer cells, consistent with previous research indicating that CAFs promote cancer cell proliferation and invasion (50). According to previous literature, we also found that the biological phenotype of ACSL4+CAFs is closer to iCAFs (51), which were associated with intra-tumoral inflammation. Inflammatory reactions within tumors can have a dual effect, recruiting more immune cell infiltration to enhance anti-tumor immunity while also leading to immune suppression due to chronic inflammation (52). It has been reported that iCAFs promote cancer cell proliferation, epithelial-mesenchymal transition (EMT), and the establishment of an immune-suppressive microenvironment (53). Research has shown that iCAFs promote immune suppression by releasing cytokines such as IL-6 and IL-10 to induce M2 polarization in macrophages (54). In our study, we found high expression of the ferroptosis marker gene ACSL4 on this subtype of CAFs, suggesting a role for ferroptosis in the growth and development of ACSL4+CAFs, which warrants further investigation. In the immune infiltration landscape, we also found a possible association between Tregs and ACSL4+CAFs, with increased infiltration of Tregs in the high ACSL4+CAFs group, providing further support for the notion of ACSL4+CAFs promoting immune suppression. Interestingly, we also discovered a noteworthy subpopulation of macrophages: FTH1+macrophages, closely associated with SPP1+macrophages. Studies have shown that SPP1+macrophages promote colorectal cancer cell proliferation and limit T cell infiltration, and their increased proportion in the TME is associated with worse patient prognosis (55, 56). FTH1+ macrophages also exhibited strong glycolysis metabolism. Some studies suggested that the metabolic reprogramming of glycolysis was crucial for macrophage polarization. We hypothesized that glycolytic activity was linked to the immunosuppressive environment in BCa and promoted the progression of BCa. In our study, we found that FTH1+macrophages were associated with chemokine-regulated signaling pathways, which might be an important mechanism through which FTH1+macrophages regulated the immune microenvironment.

Through cell-cell communication analysis, we identified SDC1 as a target mediating crosstalk between BCa cells and ACSL4+CAFs. SDC1, also known as CD138, belongs to the syndecan proteoglycan family (57). It served as an important surface adhesion molecule involved in maintaining cell morphology and interacting with the surrounding microenvironment (58). Previous literature has reported varied expression of SDC1 in different cancers (59, 60), with decreased expression in gastric and colorectal cancers but increased expression in plasmacytoid urothelial carcinoma and pancreatic cancer (61). Particularly in pancreatic cancer, its silencing can inhibit cancer progression (62). al (63). Additionally, pancreatic cancer cells expressing SDC1 can interact with T cells expressing CCL5 in the TME, promoting tumor migration, and thereby providing a potential target for immunotherapy in pancreatic cancer (64). Overall, high expression of SDC1 presented in tumors generally predicted poor prognosis due to its association with cellular component or collagen matrix. This study revealed that SDC1 could be used as a potential marker and therapeutic target for bladder urothelial carcinoma.

## Conclusion

We utilized NMF dimensionality reduction clustering to identify a novel ferroptosis-related TME cell subpopulation, ACSL4+CAFs, in BCa single-cell transcriptome data, uncovering its involvement in various phenotypes of bladder cancer. Subsequently, through integration with bulk RNA-seq data, we validated its prognostic value. Finally, cell-cell communication analysis revealed a potential target, SDC1, providing new strategies for managing BCa patients.

## Data Availability

The original contributions presented in the study are included in the article/**Supplementary Materials**, further inquiries can be directed to the corresponding authors.

## Supplementary material

Supplementary Figure 1 Trajectory analysis for ferroptosis-related CAF subpopulations. **(A)** The dot plot showed the marker genes of stromal cells. **(B)** Heatmap displaying the signaling pathways of CAF subpopulations. **(C)** Ligand-receptor analysis for CAF subpopulations. **(D)** Heatmap showed the role of ferroptosis-related marker genes in CAFs. **(E, F)** The pseudotime trajectory of CAF subpopulations.

Supplementary Figure 2 Trajectory analysis for ferroptosis-related macrophage subpopulations. **(A)** The t-SNE visualization for myeloid cells in BCa. **(B)** The dot plot showed the marker genes of myeloid cells. **(C, D)** The pseudotime trajectory analysis for macrophage subpopulations. **(E)** The cell-cell communication strength between the ferroptosis-related macrophage subpopulations and cancer cells. **(F)** Heatmap displaying the signaling pathways of macrophage subpopulations. **(G)** Ligand-receptor analysis for macrophage subpopulations. **(H)** The heatmap displayed the differential genes and enriched signaling pathways of each subpopulation.
