# Supplementary figures and images for: Integrating single-cell transcriptomics to reveal the ferroptosis regulators in the tumor microenvironment that contribute to bladder urothelial carcinoma progression and immunotherapy
Source: Front Immunol. 2024 Aug 22;15:1427124. doi: 10.3389/fimmu.2024.1427124 (PMC11375220; doi:10.3389/fimmu.2024.1427124)

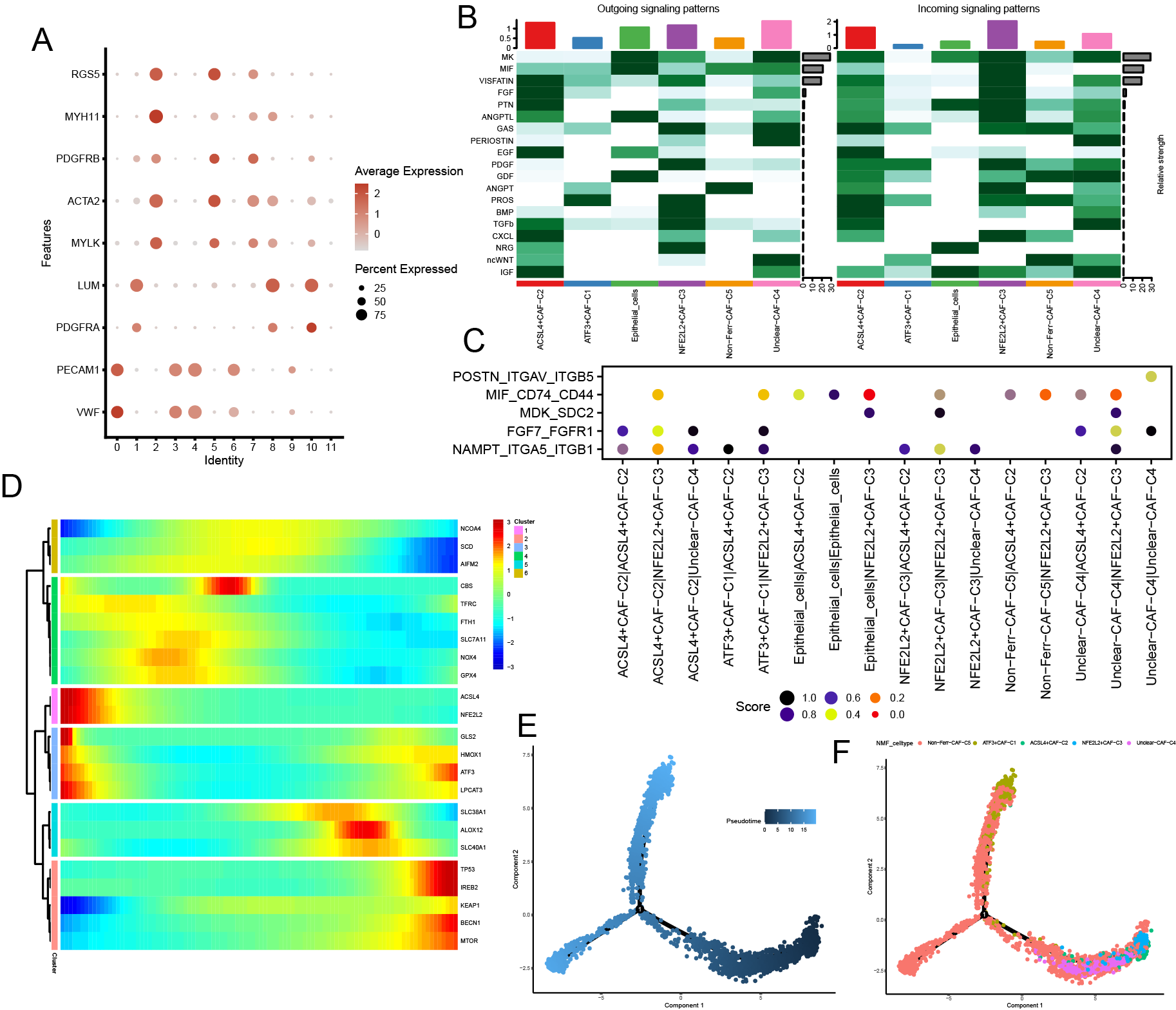

Supplement: Supplementary Figure 1 — Trajectory analysis for ferroptosis-related CAF subpopulations. (A) The dot plot showed the marker genes of stromal cells. (B) Heatmap displaying the signaling pathways of CAF subpopulations. (C) Ligand-receptor analysis for CAF subpopulations. (D) Heatmap showed the role of ferroptosis-related marker genes in CAFs. (E, F) The pseudotime trajectory of CAF subpopulations. [file Image1.tif]

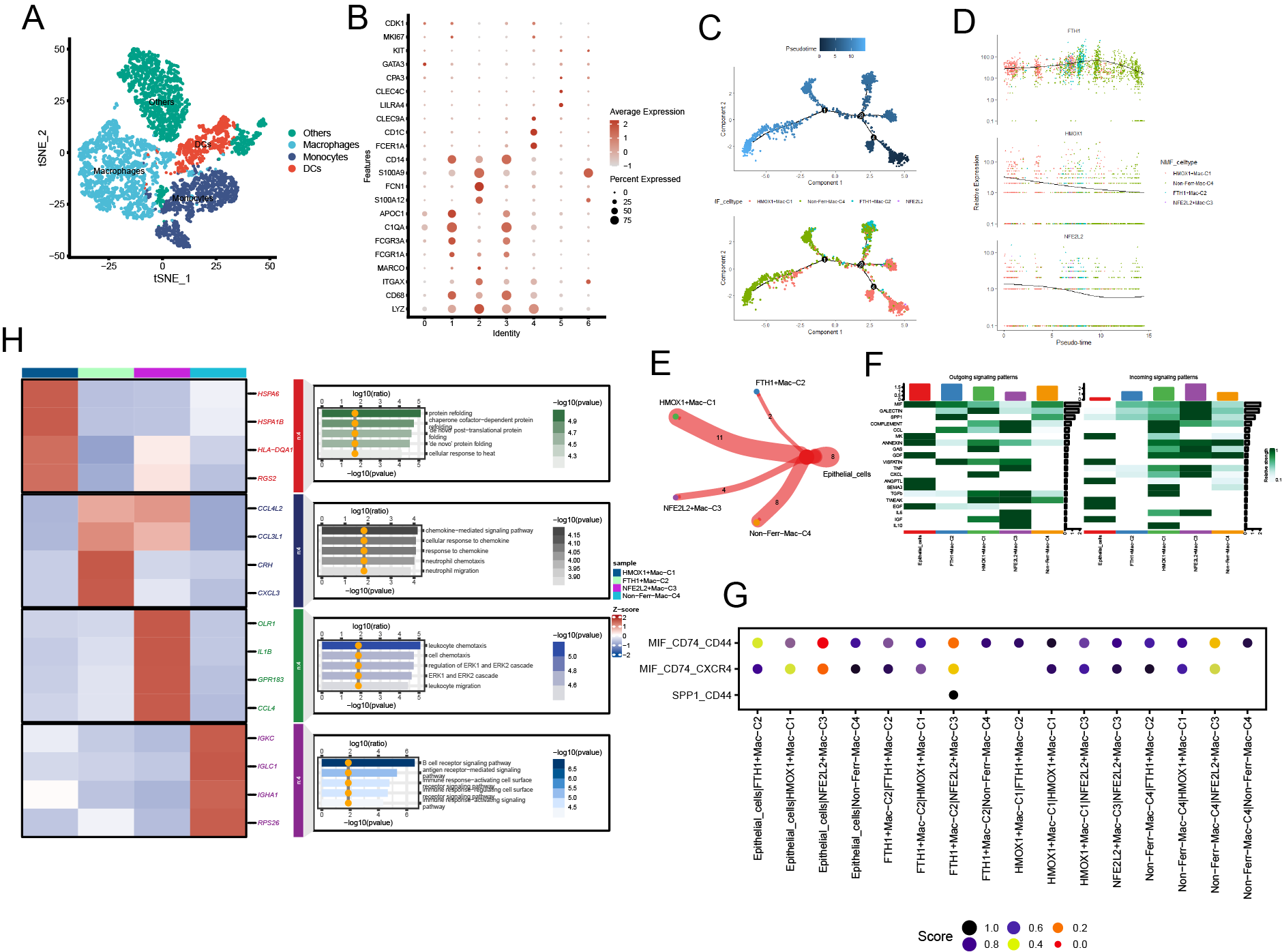

Supplement: Supplementary Figure 2 — Trajectory analysis for ferroptosis-related macrophage subpopulations. (A) The t-SNE visualization for myeloid cells in BCa. (B) The dot plot showed the marker genes of myeloid cells. (C, D) The pseudotime trajectory analysis for macrophage subpopulations. (E) The cell-cell communication strength between the ferroptosis-related macrophage subpopulations and cancer cells. (F) Heatmap displaying the signaling pathways of macrophage subpopulations. (G) Ligand-receptor analysis for macrophage subpopulations. (H) The heatmap displayed the differential genes and enriched signaling pathways of each subpopulation. [file Image2.tif]
